# The use of the vaccinia virus complement control protein (VCP) in the rat retina

**DOI:** 10.1371/journal.pone.0193740

**Published:** 2018-03-13

**Authors:** Nilisha Fernando, Riccardo Natoli, Tanja Racic, Yvette Wooff, Jan Provis, Krisztina Valter

**Affiliations:** 1 The John Curtin School of Medical Research, The Australian National University, Canberra, Australia; 2 ANU Medical School, The Australian National University, Canberra, Australia; University of Florida, UNITED STATES

## Abstract

The complement system is highly implicated in both the prevalence and progression of Age-Related Macular Degeneration (AMD). Complement system inhibitors therefore have potential therapeutic value in managing excessive activation of the complement pathways in retinal degenerations. The vaccinia virus complement control protein (VCP) has been shown to be effective as a complement inhibitor in neuroinflammatory models including traumatic brain injury and spinal cord injury. We aimed to investigate the potential of VCP as a therapeutic molecule for retinal degenerations. In this study, we investigated the effect, localisation and delivery of VCP to the rodent retina. Complement inhibition activity of VCP was tested using a hemolytic assay. Photoreceptor cell death, inflammation and retinal stress were assayed to determine if any retinal toxicity was induced by an intravitreal injection of VCP. The effect of VCP was investigated in a model of photo-oxidative retinal degeneration. Localisation of VCP after injection was determined using a fluorescein-tagged form of VCP, as well as immunohistochemistry. Finally, a copolymer resin (Elvax) was trialled for the slow-release delivery of VCP to the retina. We found that a dose equivalent to 20μg VCP when intravitreally injected into the rat eye did not cause any photoreceptor cell death or immune cell recruitment, but led to an increase in GFAP. In photo-oxidative damaged retinas, there were no differences in photoreceptor loss, retinal stress (*Gfap*) and inflammation (*Ccl2* and *C3*) between VCP and saline-injected groups; however, *Jun* expression was reduced in VCP-treated retinas. After VCP was injected into the eye, it was taken up in all layers of the retina but was cleared within 1–3 hours of delivery. This study indicates that a method to sustain the delivery of VCP to the retina is necessary to further investigate the effect of VCP as a complement inhibitor for retinal degenerations.

## Introduction

Dysregulation of the complement system, a key component of the innate immune response, is highly linked to the prevalence and progression of neurodegenerative conditions, including Age-Related Macular Degeneration (AMD) (reviewed in [1–3]). AMD is the leading cause of blindness worldwide [4], and is a disease that primarily affects the photoreceptors and retinal pigment epithelium (RPE) cells in the central retina. In the more prevalent dry form of the disease, an irreversible atrophic lesion can eventually develop over time, leading to central vision loss. It is well established that immune system activation is present during lesion expansion, including the accumulation of sub-retinal macrophages [5–10], which are associated with further photoreceptor loss [11–16]. Emerging evidence also indicates that retinal macrophages may contribute to the production of complement components in retinal degenerations, utilising rodent models of photo-oxidative stress [17, 18] and ageing [19, 20].

The complement system is made up of three activation pathways, which lead to the lysis of complement component 3 (C3) via the formation of a functional C3 convertase, a central converging event in all pathways. The eventual formation of the terminal Membrane Attack Complex (MAC) leads to the lysis of foreign or apoptotic cells [21–23]. Whilst normally under close regulation, the complement system can become over-activated under disease conditions such as AMD. Histologically, studies have demonstrated that complement system by-products (e.g. CFH, CFB, C3, C5, MAC) are present in drusen, which are sub-retinal deposits of debris that accumulate in AMD retinas [24–30]. Genome-wide association studies (GWAS) have demonstrated that a single nucleotide polymorphism (Y402H variant) in complement factor H (CFH), a critical inhibitor of the alternative complement pathway, was responsible for the onset of almost 50% of all cases of AMD [27, 31–34]. Polymorphisms in C2, complement factor B (CFB) and C3 have also been associated with AMD onset [30, 35]. Additionally, another GWAS showed a global upregulation of a number of complement genes in AMD retinas (e.g. C3, C4, C1s, CFI, SERPING1) [36].

Targeting complement activation is therefore an ideal therapeutic strategy, to reduce photoreceptor loss resulting from complement deposition and MAC formation. Although a number of complement inhibitors are currently being trialled for the treatment of AMD and other retinal diseases [37], there is an unmet need for effective inhibitors to broadly target all complement pathways. As a degree of redundancy exists between the complement pathways, blocking just one pathway may not be sufficient [38]. A positive effect of the C3 inhibitor POT-4/Compstatin for AMD, having finished Phase I clinical trials, is yet to be determined. Use of the vaccinia virus complement control protein (VCP) for complement inhibition has been documented in several neuroinflammatory models (reviewed in [39]). VCP is a 35kDa immune-evasion protein isolated from the vaccinia virus which has binding sites for C3 and C4, blocking the formation of a functional C3 convertase [40]. It inhibits complement produced through both the classical and alternative pathways. Although VCP is effective at inhibiting complement and associated tissue damage in other disease models, including atherosclerosis [41], traumatic brain injury [42] and spinal cord injury [43], the effects of VCP on the eye and retina are not known.

Our laboratory has previously utilised chemokine inhibitors [44, 45] and small-interfering RNA (siRNA) [46, 47] to slow macrophage recruitment into the damaged retina, in a model of progressive photo-oxidative damage in which complement production by macrophages is a key feature [17, 46, 48]. The aim of the study was to assess the suitability of the complement inhibitor VCP as a candidate for the treatment of retinal degeneration using the photo-oxidative damage model. Here, we investigate the effect, localisation and delivery of VCP to the rodent retina.

## Materials and methods

### Animal handling and photo-oxidative damage

All experiments conducted were in accordance with the ARVO Statement for Use of Animals in Ophthalmic and Vision Research, and with approval from the Australian National University Animal Experimentation Ethics Committee (Ethics ID: A2012/07). Adult Sprague-Dawley (SD) and Wistar albino rats from the Australian Phenomics Facility aged between 120–150 post-natal days and held under dim light conditions (5 lux) were utilised throughout the study. To induce retinal degeneration, animals were exposed to 1000 lux light for 24 hours to induce photo-oxidative damage as described previously [48]. Animals were euthanized for tissue collection at 0 days (immediately after light exposure) or at 7 days post-exposure (returned to 5 lux dim conditions).

### Hemolytic assay for complement activity

The ability of VCP to inhibit complement was tested using a hemolytic assay, according to a previously described protocol [49]. In brief, sheep red blood cells (RBCs; Applied Biological Products Management, SA, Australia) were sensitised with a hemolysin antibody (Sigma-Aldrich, MO, USA). Recombinant VCP (GeneBalance Inc., MA, USA) was reconstituted using 0.9% NaCl sterile saline (Pfizer, NY, USA) to varying concentrations (0–0.6μg/μl). The antibody-sensitised red blood cells were then incubated with rabbit serum (Applied Biological Products Management) and VCP for 1 hour at 37°C. After centrifugation of the cell suspension, the supernatant was transferred to a 96-well plate in duplicate. The absorbance of hemoglobin at 540nm was measured using a spectrophotometer, and the % hemolysis of the red blood cells for each concentration of VCP was determined. Two controls were used for comparison of hemolytic activity; a 100% lysis control (RBCs with serum, no VCP) and a 0% spontaneous lysis control (RBCs only, no serum).

### Intravitreal injections and tissue collection

Using a dose of 20μg per eye, the VCP protein was reconstituted in 0.9% NaCl (saline) to a concentration of 4μg/μl. Intravitreal injections were carried out using a protocol described previously [44]. Both eyes of each animal were intravitreally injected with either 5μl VCP in saline, or 5μl saline only. Animals were left for 7 days in dim conditions, or were exposed to photo-oxidative damage for 24 hours following the injections and recovery from anaesthesia. After photo-oxidative damage, animals were immediately euthanized with an intraperitoneal overdose of barbiturate (Valabarb, Virbac, NSW, Australia). One eye of each animal was enucleated, fixed in 4% paraformaldehyde, and stored in 15% sucrose overnight prior to embedding in Tissue-Tek OCT Compound (Sakura Finetek, CA, USA). The retina was excised from the other eye and stored in chilled RNAlater for RNA extraction. Eyes were then cryosectioned at 16μm. Non-injected eyes/retinas from dim-reared control animals were collected for comparison.

### RNA extraction, cDNA synthesis and qPCR

RNA extraction was carried out using a combination of TRIzol (Thermo Fisher Scientific) and an RNAqueous Micro Total RNA Isolation Kit (Thermo Fisher Scientific) as previously described [17]. cDNA was prepared using a Tetro cDNA Synthesis Kit (Bioline, London, UK) according to the manufacturer’s instructions. Quantitative real-time polymerase chain reaction (qPCR) was carried out using Taqman hydrolysis assays (Table 1) and Taqman Gene Expression Master Mix (Thermo Fisher Scientific). Reactions were carried out on a QuantStudio Flex 12K instrument (Thermo Fisher Scientific) at the Biomolecular Resource Facility (JCSMR, ANU). The expression change of all target genes was compared to the *Gapdh* reference gene using the comparative C_t_ method (∆∆C_t_).

**Table 1:** Taqman hydrolysis assays used for qPCR.

Gene Symbol	Gene Name	Catalogue Number	Entrez Gene ID
*C3*	Complement component 3	Rn00566466_m1	24232
*Ccl2*	Chemokine (C-C motif) ligand 2	Rn01456716_g1	25542
*Gapdh*	Glyceraldehyde-3-phosphate dehydrogenase	Rn99999916_s1	24383
*Gfap*	Glial fibrillary acidic protein	Rn00566603_m1	24387
*Jun*	Jun proto-oncogene	Rn99999045_s1	24516

### TUNEL and immunohistochemistry

Retinal sections were assayed in duplicate per animal for photoreceptor cell death using a terminal deoxynucleotidyl transferase dUTP nick end labelling (TUNEL) kit (Roche, Basel, Switzerland) according to previously described protocols [50, 51]. TUNEL+ photoreceptors in the outer nuclear layer (ONL) were counted, or ONL thickness measured at the lesion site (2mm superior to the optic nerve) using bisbenzimide (1:10000; Sigma-Aldrich) labelling and an LSM 5 PASCAL confocal microscope (Zeiss, West Germany). Relative ONL thickness was calculated as a ratio of the ONL thickness to the distance between the inner and outer limiting membranes. To detect microglia and macrophages in the retina, immunohistochemistry using an α-IBA1 primary antibody (1:500, #019–19741, Wako, Osaka, Japan) was performed on retinal sections according to previously described protocols [52, 53]. IBA1+ cells in the outer retina (ONL-RPE) were counted for each retinal section. To detect retinal stress, immunohistochemistry using an α-GFAP primary antibody (1:500, #Z033429-2, Agilent Technologies, CA, USA) was performed on retinal sections. Fluorescence intensity for GFAP labelling was measured using ImageJ (NIH, MD, USA).

### Detection of VCP in the retina using fluorescein

The fluorescein-labelled VCP was prepared using an NHS-Fluorescein Antibody Labelling Kit (Pierce, Thermo Fisher Scientific, MA, USA). In brief, the VCP protein was reconstituted in 0.9% NaCl (saline) to a concentration of 2μg/μl. The VCP was then incubated with the NHS-Fluorescein for 1 hour, and was stored at 4°C until required for the injections. Animals were injected with 5μl fluorescein-VCP into one eye, whilst the fellow eye served as a non-injected control. Eyes were then collected at several time points (20mins, 1hr, 3hrs, 6hrs, 14hrs and 24hrs) after the injection, and were sectioned. Fluorescein-VCP was visualised in retinal sections under the confocal microscope.

### Detection of VCP in the retina using immunohistochemistry

To verify the localisation of VCP in the retina in addition to fluorescein labelling, an α-VCP (anti-SPICE hybridoma KL5.1, 1.64mg/ml) mouse monoclonal antibody [54] was utilised to detect VCP in retinal sections. Specificity of the α-VCP antibody was verified using dot blot analysis. In summary, the VCP protein was transferred onto a 0.22μm PVDF membrane. A mixture of isolated proteins from the retina was also blotted onto the membrane as a control. The membrane was then blocked in 3% bovine serum albumin (BSA) for 3 hours before being incubated with the α-VCP antibody (1:100) overnight at 4°C. The next day, the membrane was incubated with a HRP-conjugated secondary antibody (BioRad, CA, USA) for 2 hours, and a peroxidase colour substrate (Vector VIP; Vector Labs, CA, USA) was added to visualise the reaction. Dots on the membrane were imaged using the confocal microscope. Immunohistochemistry was performed on retinal sections using the α-VCP antibody (1:100) according to previously described protocols [52, 53].

### Delivery of VCP using Elvax

To test the slow-release delivery of VCP using an Elvax (ethylene vinyl acetate) copolymer resin implant in the eye, a VCP-Elvax drug-resin complex was prepared according to previously described protocols [55]. In brief, 40μl of fluorescein-VCP (2μg/μl) was incorporated into the Elvax resin to form a drug-resin complex. After freeze-drying the resin to form a block, a fluorescein-VCP-Elvax implant was prepared from the block, and was implanted into one eye of each animal, following previously published procedures [55]. The contralateral eye was intravitreally injected with 5μl fluorescein-VCP (2μg/μl) as described previously. Eyes were collected at several time points (20mins, 6hrs, 24hrs and 7 days) after the implantation or injection and were sectioned. Fluorescein-VCP was visualised in retinal sections under the confocal microscope.

### Imaging and statistical analysis

For each retinal section, images of fluorescence in the superior retina (2mm from the optic nerve) were acquired using an LSM 5 PASCAL confocal microscope (Zeiss, West Germany) or an A1^+^ laser-scanning confocal microscope (Nikon, Tokyo, Japan). Images were analysed and processed using ImageJ and Photoshop CS6 software (Adobe Systems, CA, USA). All graphing and statistical analysis was performed using Prism 6 (GraphPad Software, CA, USA), using unpaired Student’s t-tests, or 1-way analysis of variance (ANOVA) with Tukey’s multiple comparisons post-test to determine statistical significance (*P<0.05).

## Results

### Effective dose of VCP required for complement inhibition

**Fig 1:**
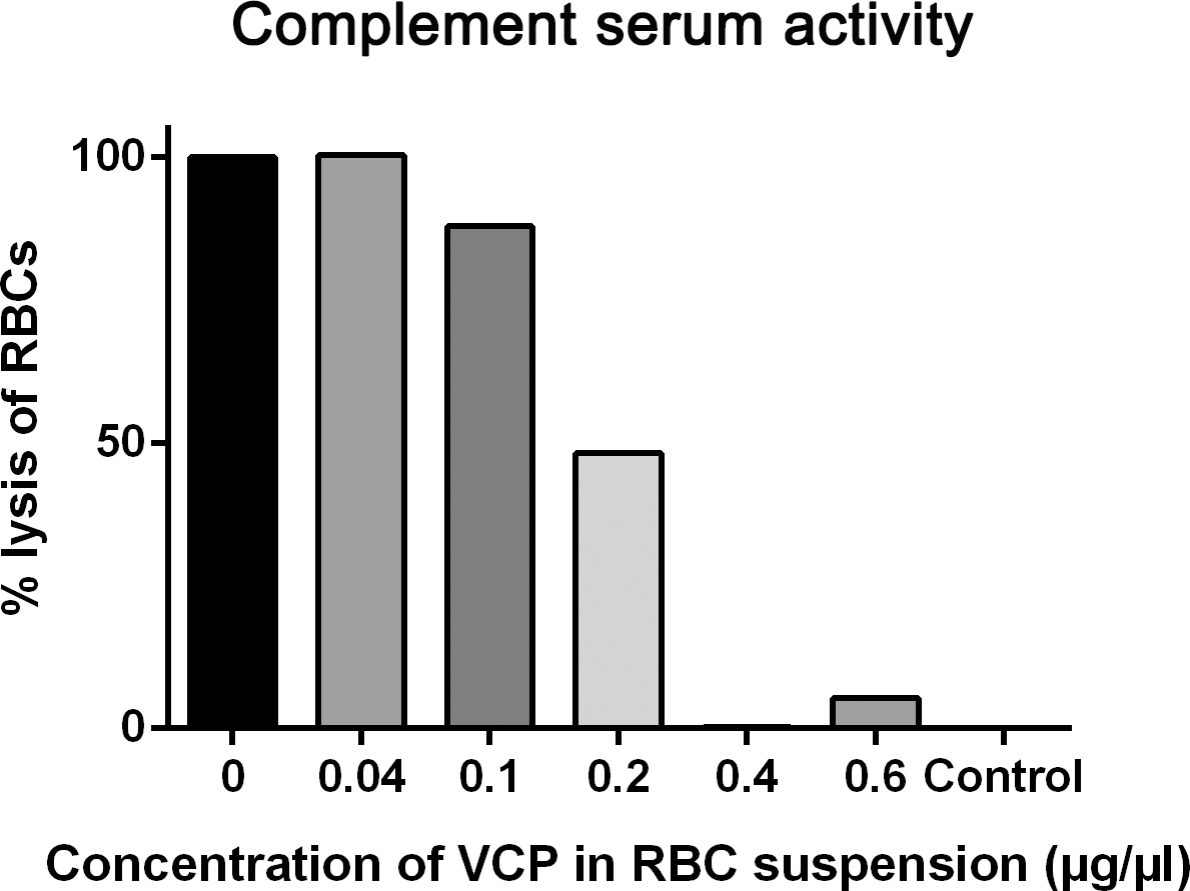
Complement activity of rabbit serum when incubated with sheep red blood cells (RBCs) as determined via hemolytic assay. Varying concentrations of VCP (0–0.6μg/μl) were added to each RBC suspension. 100% lysis of RBCs was measured when only serum (no VCP) was added. The spontaneous lysis control (no serum) had 0% lysis. VCP inhibited at least 50% RBC lysis at concentrations between 0.2–0.4μg/μl.

To establish an effective dose of VCP for complement inhibition, complement activity of rabbit serum was tested in a hemolytic assay, where varying concentrations of VCP (0–0.6μg/μl) was added. The % lysis of RBCs was determined for each concentration of VCP in the cell suspension, which was 100% when only serum (no VCP) was added, indicating maximal complement activity of the serum (Fig 1). The spontaneous lysis control (no serum) had 0% lysis. Addition of 0.1μg/μl VCP caused a reduction of hemolysis by approximately 10%, whereas addition of 0.2μg/μl VCP decreased hemolysis by approximately 50%, demonstrating increasing complement inhibition relative to additional VCP. By addition of 0.4μg/μl VCP, there was a near-complete inhibition of complement serum activity. This indicates that a concentration of 0.2–0.4μg/μl VCP was effective at inhibiting complement. This dose is equivalent to 20μg in the rat eye, in which the total vitreal volume is 50–55μl.

### Assessment of safety of VCP in the retina

**Fig 2:**
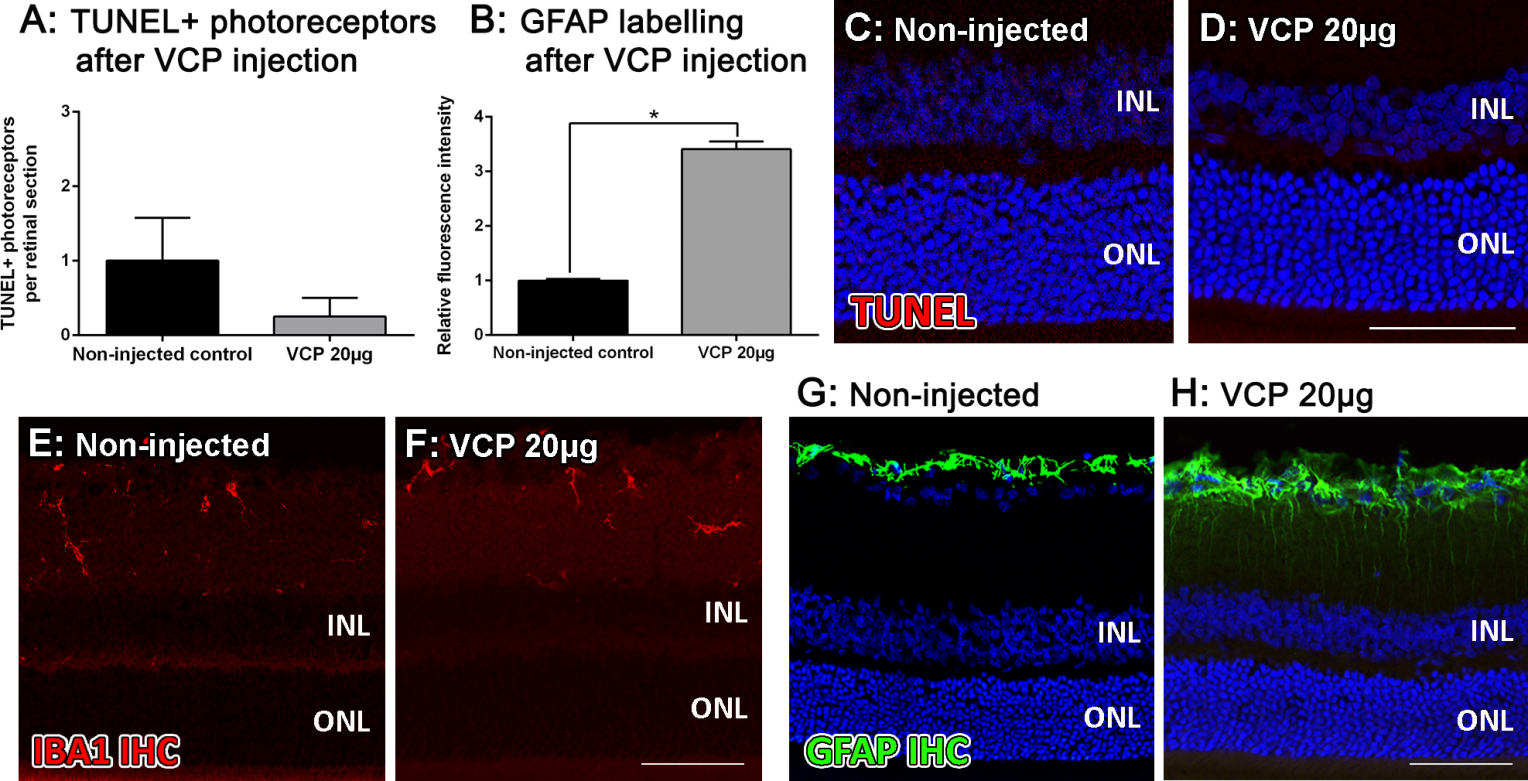
Effect of an intravitreal injection of 20μg VCP on cell death, inflammation and retinal stress at 7 days. **A:** There was no significant difference in the number of TUNEL+ photoreceptors between VCP-injected and non-injected retinas (P>0.05). B: A significant increase in GFAP labelling was detected after the VCP injection compared to non-injected controls (P<0.05) **C-D:** No TUNEL+ cells were observed in the inner retina, and only 0–1 TUNEL+ photoreceptors per retinal section were identified in both VCP-injected and non-injected groups. No difference in retinal morphology was observed between these groups. **E-F:** IBA1+ cells remained in the inner retina and displayed a ramified morphology. No IBA1+ cells were detected in the outer retina of either group. **G-H:** Low levels of GFAP immunostaining was observed near the GCL, indicating Müller cell and astrocyte labelling in the non-injected control (G), however an increase in GFAP was detected in the VCP-injected retina (H). INL, inner nuclear layer; ONL, outer nuclear layer. Statistical analysis was performed using a Student’s t-test. Scale bars indicate 50μm and error bars are displayed as SEM. N = 3 for all experimental groups.

To explore any toxicity that may arise from delivering 20μg VCP in 5μl saline to the rat eye, retinal histology was assessed using TUNEL as a measure of photoreceptor cell death, bisbenzimide to visualise the retinal layers, IBA1 immunohistochemistry to quantify the recruitment of retinal microglia/macrophages to the outer retina (ONL-RPE), and GFAP immunohistochemistry to determine general retinal stress. At 7 days after an intravitreal injection of 20μg VCP in 5μl saline to the rat eye, there was no significant difference in the number of TUNEL+ photoreceptors compared to non-injected controls, which were only 0–1 cells per retinal section (P>0.05, Fig 2A, 2C and 2D). There was no visible difference in photoreceptor layer organisation, or overall retinal histology between VCP-injected and non-injected groups (Fig 2C and 2D). No IBA1+ cells were detected in the outer retina of both VCP-injected and non-injected groups at 7 days, indicating little to no retinal inflammation following the intravitreal injection (Fig 2E and 2F). However, at 7 days after VCP was injected, there was a significant increase in GFAP staining in the VCP-injected retinas compared to non-injected controls, indicating that mild stress was induced in the retina following the VCP injection (P<0.05, Fig 2B, 2G and 2H).

### Effect of VCP on the photo-oxidative damaged retina

**Fig 3:**
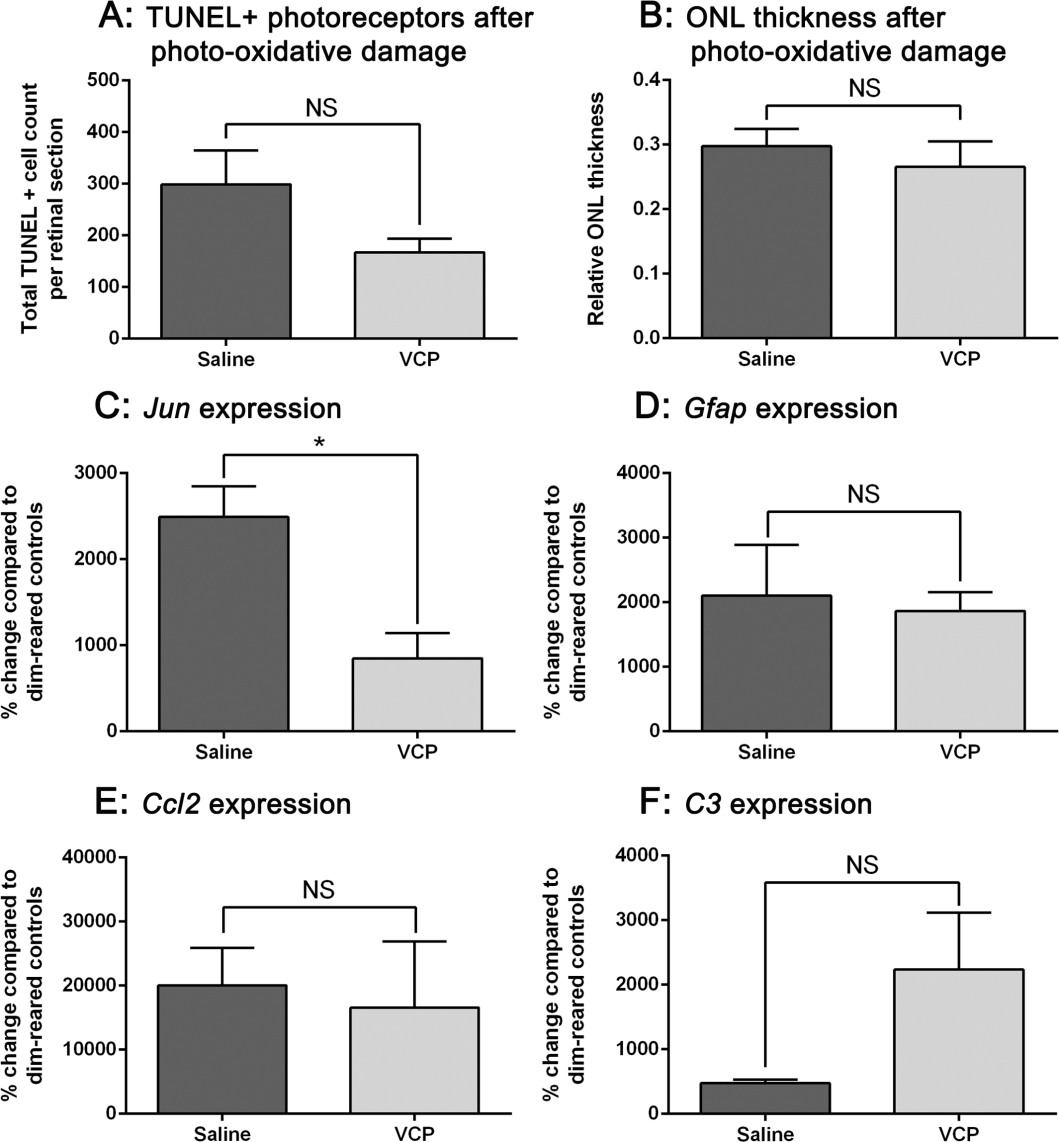
Effect of an intravitreal injection of 20μg VCP on the photo-oxidative damaged retina. **A:** Immediately after photo-oxidative damage, there was a reduction in TUNEL+ photoreceptors in the VCP-injected retinas compared to the saline-injected retinas, however this was not significant (P>0.05). **B:** At 7 days after photo-oxidative damage, there was no significant change in outer nuclear layer (ONL) thickness at the lesion site (2mm superior from the optic nerve) between the VCP and saline groups. **C:** After photo-oxidative damage, *Jun* gene expression was decreased significantly in VCP-injected retinas compared to saline controls (P<0.05). **D-F:** Retinal expression of *Gfap*, *Ccl2* and *C3* did not significantly change between the VCP and saline groups following photo-oxidative damage (P>0.05). * indicates a statistical significance of P<0.05. Statistical analysis was performed using a Student’s t-test. Scale bars indicate 50μm and error bars are displayed as SEM. N = 10–16 (A), N = 4 (B), N = 3 (C-F) for each experimental group.

We assessed the effect of delivering VCP to the retina prior to photo-oxidative damage. Animals were intravitreally injected with 20μg VCP or saline immediately prior to exposure to photo-oxidative damage for 24 hours, and eyes were collected both immediately and at 7 days after light exposure. Following retinal damage, there was a trend towards a reduction in photoreceptor cell death in the VCP-injected retinas compared to the saline-injected controls; however, this change was not significant (P>0.05 Fig 3A). Outer nuclear layer (ONL) thickness was measured at the lesion site (2mm superior to the optic nerve in retinal cryosections) at 7 days after photo-oxidative damage to investigate any cumulative photoreceptor cell loss that may have occurred. There was no significant difference in photoreceptor layer thickness between the two groups (P>0.05, Fig 3B). Changes in whole retinal gene expression was determined using qPCR. There was a significant reduction in *Jun*, a marker of apoptosis, in VCP-injected retinas compared to saline controls (P<0.05, Fig 3C). There was no significant change in the retinal stress marker *Gfap*, or inflammatory markers *Ccl2* and *C3* in the retina between VCP and saline groups (P>0.05, Fig 3D and 3F).

### Localisation of VCP in the retina

**Fig 4:**
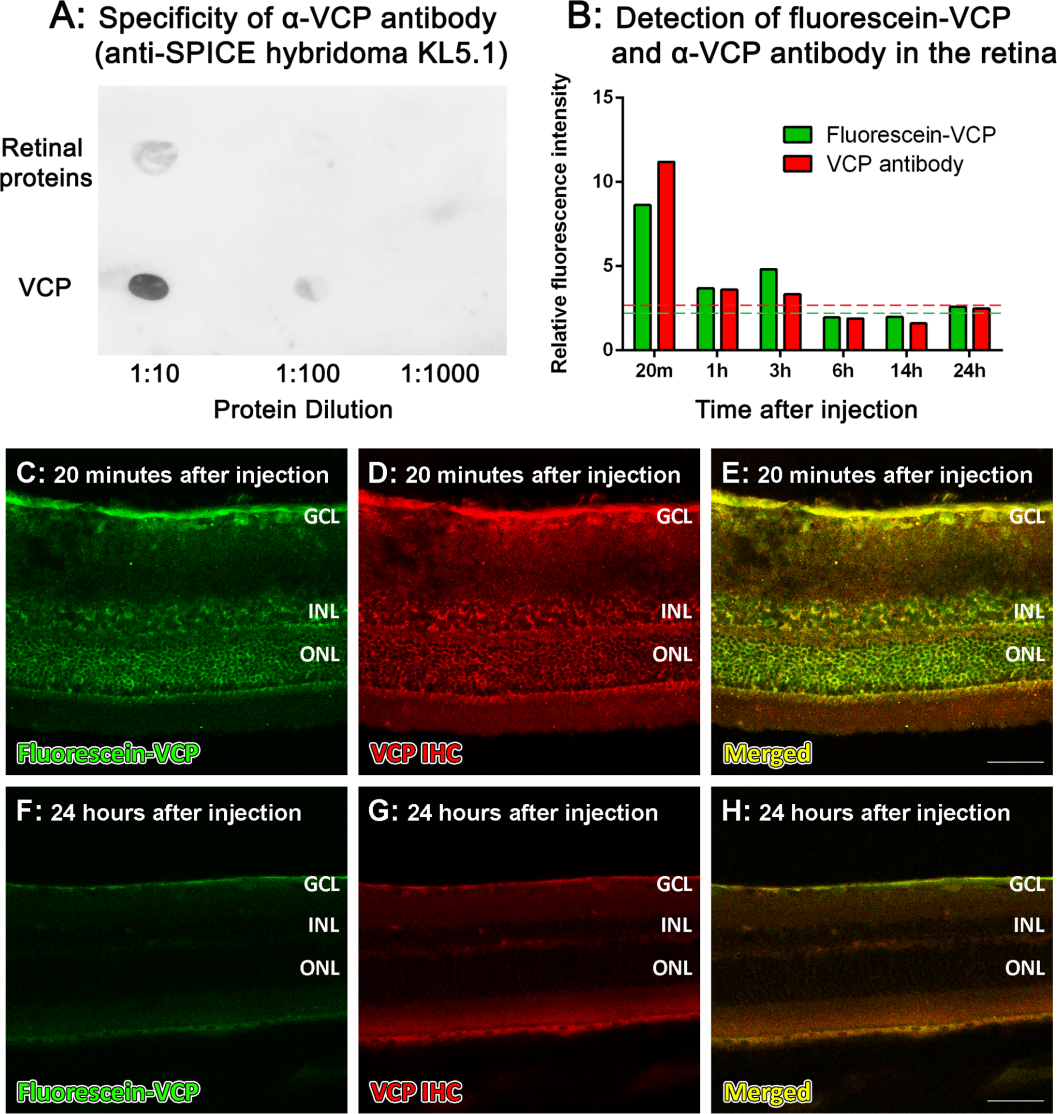
Localisation of VCP in the retina following an intravitreal injection. **A:** Dot blot analysis showed immunoreactivity of the α-VCP antibody (anti-SPICE hybridoma KL5.1) for VCP, compared to mixed retinal proteins (control). **B-H:** 10μg of fluorescein-VCP was intravitreally injected into the retina, and retinal fluorescence intensity (B) was determined from 20 minutes to 24 hours after injection. The red dashed line represents background red fluorescence levels in negative control and non-injected control sections, and the green dashed line represents background green fluorescence levels of non-injected control sections. Fluorescein-VCP was detected in all layers of the retina, and was evenly distributed from the central retina to the periphery at 20 minutes (C). However, at 1–3 hours after injection, the fluorescein-VCP was cleared from the retina, which remained at background levels of fluorescence up to 24 hours (F). Double labelling with the α-VCP antibody was used to verify localisation of VCP in the retina (B, D, E, G, H). GCL, ganglion cell layer; INL, inner nuclear layer; ONL, outer nuclear layer; h, hours; m, minutes. Scale bars indicate 50μm.

We investigated the retinal localisation of VCP following an intravitreal injection, where 10μg of fluorescein-VCP was injected into each eye (concentration at 2mg/ml optimised for the fluorescein labelling kit). At 20 minutes after the injection, fluorescein-VCP was detected in all layers of the inner and outer retina (Fig 4B and 4C). This was observed in nasal, central and temporal parasagittal sections, and the fluorescein-VCP was distributed evenly from the central retina to the peripheral retina. However, from 1–3 hours after injection onwards (Fig 4B), fluorescein-VCP was cleared from the retina, and only background levels of fluorescence were detected up until 24 hours (Fig 4F). An α-VCP antibody, confirmed to have immunoreactivity for VCP (Fig 4A), was used to verify VCP localisation in the retina, which co-localised at all time points with the fluorescein-VCP (Fig 4B, 4D, 4E, 4G and 4H). This data indicates that VCP is cleared from the retina within 1–3 hours after intravitreal delivery.

### Slow-release delivery of VCP using Elvax

**Fig 5:**
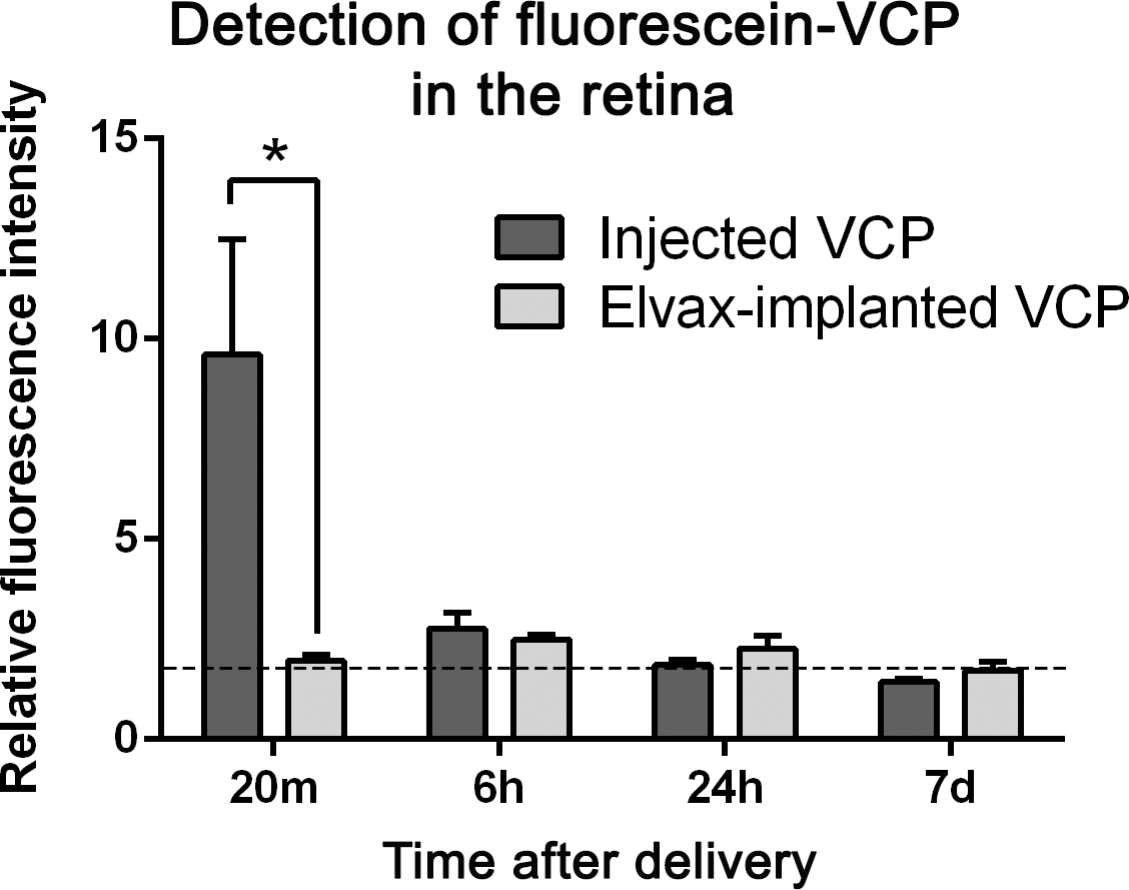
Quantification of fluorescein-VCP in the retina following an intravitreal injection, or delivery through an implanted Elvax complex. At 20 minutes after the VCP-Elvax was delivered to the eye, there was significantly less fluorescein-VCP in the retina than after the intravitreal injection (P<0.05). At 6 hours, 24 hours and 7 days, only background levels of fluorescence were evident in both the injected and implanted eyes (dashed black line represents average background in non-injected control sections). Statistical analysis was performed using a 1-way ANOVA with Tukey’s multiple comparisons post-hoc test. * indicates a statistical significance of P<0.05. d, days; h, hours; m, minutes. Scale bars indicate 50μm and error bars are displayed as SEM. N = 3 for all experimental groups.

To provide an extended drug delivery of VCP to the retina, a slow-release delivery vehicle (Elvax) was used, where the drug-resin complex is inserted into the eye [55]. Fluorescein-VCP was delivered through this complex, and fluorescence intensity was measured across a time course. While we observed fluorescein-VCP release from the complex, we were unable to find levels of VCP in retinal cryosections above background (Fig 5, dashed black line). At 20 minutes, fluorescein-VCP intravitreally injected into the eye was present in all layers of the retina as observed previously in Fig 4. The contralateral eye, which contained the VCP-Elvax complex had only background levels of fluorescence at 20 minutes, which was significantly less than the injected eye (P<0.05, Fig 5). Eye injected with fluorescein-VCP and implanted with VCP-Elvax had only background levels of fluorescence in the retina at 6 hours, 24 hours and 7 days after the drug was delivered, indicating that minimal VCP was detected in the retina at each time point following Elvax drug delivery.

## Discussion

This study aimed to investigate the use of the complement inhibitor VCP in the eye for the treatment of retinal degenerations, where excessive complement activation is a key feature of disease progression. We demonstrated that a concentration of 0.2–0.4μg/μl VCP is effective at inhibiting complement, which is equivalent to a dose of 20μg VCP in the rat eye. This dose did not induce any cell death or inflammation when delivered intravitreally to the eye, however led to an increase in GFAP levels, indicating mild retinal stress. In photo-oxidative damaged retinas, there was no significant change in photoreceptor loss, and expression of retinal stress (*Gfap*) and inflammation (*Ccl2* and *C3*) between VCP and saline-injected groups. However, *Jun* expression was significantly reduced in VCP-injected retinas compared to saline controls. We found that after delivering VCP to the eye, it was cleared from all layers of the retina within 1–3 hours. The findings indicate that if VCP is to be pursued as a candidate for the treatment of retinal degenerations, a slow-release delivery vehicle or method to extend the life of VCP in the eye is necessary.

VCP is a novel C3 inhibitor known to carry out complement inhibition by acting on the C3 convertases of all pathways, preventing C3 from being hydrolysed [40]. In the current study, we have shown via a hemolytic assay that complement serum activity is inhibited following incubation with 0.2–0.4μg/μl VCP. The equivalent dosage in the rat eye is approximately 20μg VCP, which would vary between rats, mice and humans due to differences in vitreal volume. In the rat retina, this dose was found to be non-toxic when delivered intravitreally, causing negligible photoreceptor cell death. In previous studies using various neuroinflammatory disease models, a similar dose of VCP has been administered. In a model of traumatic brain injury, 22.5μg VCP was injected into the brain which improved spatial memory compared to saline-treated controls [42]. Another study found that two bilateral intraparenchymal injections of 24μg VCP was able to inhibit macrophage accumulation and improve function in a model of spinal cord injury [43]. It is indicated that this dose of VCP may be effective against neuroinflammation caused by retinal degenerations.

The key role of the complement system in the progression of retinal degenerations including AMD is well-established [3]. In the rodent photo-oxidative damage model that recapitulates several features of the atrophic form of AMD [48, 56], excessive complement activation is known to be linked to a progressive loss of photoreceptors within a focal retinal lesion [46]. This complement activation is associated with activated microglia and macrophages, which are recruited to the lesion site and are known to produce complement components that may lead to further photoreceptor cell death [17, 46]. We wanted to investigate whether the effect of VCP could be tested using the photo-oxidative damage model. There were no significant differences in photoreceptor loss and the expression of *Gfap*, *Ccl2* and *C3* between VCP and saline-injected groups.

In contrast, *Jun* gene expression was significantly decreased in the VCP-treated retinas compared to saline controls. *Jun* encodes the transcription factor AP-1 involved in apoptosis-related mechanisms. Together, with the trend towards a reduction in photoreceptor cell death immediately after photo-oxidative damage in the VCP-treated retinas, it is indicated that there could be a decrease in photoreceptor death, possibly through complement inhibition. However, at 7 days after photo-oxidative damage, VCP had no effect on photoreceptor loss. In our previous studies, we have shown that the delivery of *C3* siRNA to the retina can ameliorate complement-mediated photoreceptor loss over time [46], demonstrating that C3 inhibition as a therapeutic strategy may be beneficial. Although VCP has the ability to control complement, our present data suggest that its half-life in the retina is very short, and consequently does not lead to the expected outcomes *in vivo*.

Delivery of fluorescein-tagged VCP to the eye indicated that VCP was taken up by all layers of the retina, but cleared from the retina within 1–3 hours. The mechanism for the rapid clearance of VCP from the retina is not known. It may be cleared through the vitreous via the anterior chamber, or alternatively across the blood-retinal barrier in the posterior chamber. Compounds with a larger molecular weight and increased water-solubility tend to have a longer half-life in the vitreous [57]. Although VCP is water-soluble, its smaller molecular weight might contribute to its fast clearance from the eye. When delivered intraperitoneally at a concentration of 20mg/kg [41], VCP activity in serum lasts 3–4 days. Further, it is thought that heparin-binding sites of VCP may allow for sequestering of the compound in specific tissues, thereby extending its half-life [40, 58, 59]. The possibility for heparin-binding as a mechanism for VCP’s rapid clearance may warrant further investigation.

We also investigated the use of a VCP-Elvax implant, to provide extended release of fluorescein-labelled VCP into the retina. This technique has been used previously to effectively slow-release compounds into the eye, with a functional effect on the retina [55]. While we observed the release of fluorescein-VCP from the implant into the eye in tissue sections, we did not detect an increase in fluorescein-VCP in the retinal layers. It is likely that small amounts of VCP were being released from the complex into the eye, but were cleared from the retina resulting in minimal detection of the compound.

Other particle carriers including biodegradable microspheres, nanoparticles and liposomes have proven efficacious at delivering a sustained release of drugs to the eye [60–62], but may not extend the availability of VCP in the retina itself beyond 1–3 hours. As our photo-oxidative retinal degeneration model requires 24 hours of light exposure, which induces photoreceptor cell death, inflammation and complement over the ensuing 7 days [46, 48], the half-life of VCP in the retina needs to be extended to appropriately assess its effect against retinal damage. Further investigation into prolonging the availability of VCP in the retina itself is necessary before VCP can be considered further than a theoretical candidate for use in models of retinal degeneration.
